# Comparatıve evaluatıon of the brıgantı nomogram, partın nomogram, mskcc nomogram and a machıne learnıng-based model ın predıctıng lymph node ınvasıon before radıcal prostatectomy

**DOI:** 10.1186/s12894-026-02128-y

**Published:** 2026-04-01

**Authors:** Ismail Onder Yılmaz, Ozgür Yılmaz, Mehmet Zubaroglu, Rahmi Kavak, Nebil Akdogan, Mehmet Gurkan Arikan, Mutlu Deger, Volkan Izol

**Affiliations:** 1 Department of Urology, Faculty of Medicine, , Cukurova Unıversity, Adana, Turkey; 2 Department of Artificial Intelligence Engineering, Alparslan Turkes Science and Technology University, Adana, Turkey; 3 Department of Urology, Tarsus State Hospital, Mersin, Turkey; 4 Department of Software Engineering, Alparslan Turkes Science and Technology University, Adana, Turkey; 5 Private Clinic, Cukurova Urology Center, Adana, Turkey

**Keywords:** Lymph Node Invasion, Prostate Cancer, Machine Learning, Nomograms, Predictive Accuracy, Decision Support

## Abstract

**Introduction:**

Accurate preoperative prediction of lymph node invasion (LNI) is crucial for deciding on extended pelvic lymph node dissection (ePLND) in radical prostatectomy. Traditional nomograms such as Briganti, Partin, and MSKCC are widely used, but machine learning (ML)–based models may improve predictive accuracy.

**Materials and methods:**

Data from 471 prostate cancer patients were analyzed, including demographic, clinical, and histopathological variables and scores from Briganti, Partin, and MSKCC nomograms. Eleven ML algorithms were evaluated, with performance assessed by AUC-ROC, accuracy, sensitivity, specificity, and F1-score. Class imbalance was addressed with resampling techniques, and feature importance analyses were performed.

**Results:**

LNI was present in 97 patients (20.6%). Significant predictors included MSKCC and Partin scores, PSA, ISUP grade, PIRADS score, lymphovascular invasion, and age. Neural Network (AUC: 0.81) and Random Forest (AUC: 0.77) showed similar performance to the nomograms (MSKCC: 0.79; Briganti: 0.77; Partin: 0.78) when considering the AUC values and their 95% confidence intervals.Decision tree analysis highlighted negative core count, ISUP grade, prostate density, PSA, BMI, and age as key variables. Combining nomogram scores with ML models resulted in numerically slightly higher AUC values; however, these differences remained within a similar performance range and did not indicate a clinically meaningful improvement.

**Conclusion:**

Although the AUC values of the ML models appear numerically comparable to, or slightly higher than, those of traditional nomograms, the inherent limitations of the study preclude demonstrating a clinically superior or reliably advantageous performance; therefore, multicenter prospective validation studies are warranted.

## Introduction

Prostate cancer (PCa) is one of the most common malignancies in men and the second most frequently diagnosed cancer worldwide [1, 2].

According to the 2025 European Association of Urology (EAU) guidelines, extended pelvic lymph node dissection (ePLND) is recommended for high-risk and selected unfavourable intermediate-risk patients when the predicted probability of lymph node invasion is generally ≥ 5% or otherwise clinically significant [3].

Whether ePLND provides a clear therapeutic benefit remains a key clinical question in prostate cancer surgery. Contemporary 2025 evidence indicates that prostate-specific membrane antigen positron-emission tomography/computed tomography (PSMA PET/CT) alone is insufficient to determine the need for pelvic lymph-node dissection and should be integrated with clinical and imaging-based risk models of lymph-node invasion [4]. Recent literature has reported updated nomogram studies incorporating PSMA-PET imaging, demonstrating that the inclusion of PSMA-PET findings into existing nomograms significantly improves the accuracy of lymph node invasion prediction and meaningfully reduces the false-negative rate of ePLND [5]. In line with these observations, the Amsterdam–Brisbane–Sydney (ABS) nomogram has also shown that integrating PSMA-PET data into risk calculations provides superior predictive performance compared to conventional models and has been successfully validated across diverse geographic populations [6]. Moreover, in patients who are preoperatively negative for pelvic nodal involvement on PSMA imaging, externally validated nomograms have been shown—around the 5% decision threshold—to reduce unnecessary ePLND’s while maintaining oncologic safety [7]. Externally validated nomograms—such as the Briganti nomogram developed based on clinical stage, serum PSA levels, Gleason score, and biopsy findings—are used to calculate this risk. Patients with ISUP Grade Group 3, especially those with high Gleason scores and elevated PSA levels, should be evaluated using advanced imaging methods (abdominopelvic CT/MRI and bone scintigraphy). In these patients, who carry a high risk of lymph node metastasis, ePLND is recommended. Conversely, LND is not recommended for patients with a lymph node invasion risk below 5%, as the potential surgical morbidity is considered to outweigh the possible clinical benefits in this group [8].

Accurate identification of patients at risk for lymph node invasion (LNI) is critically important to avoid unnecessary procedures, protect patients from potential surgical complications, and improve treatment outcomes, given that lymph node dissection (LND) is associated with potential comorbidities [9]. To this end, commonly used risk-prediction tools include the Briganti [10, 11], Partin [12], and MSKCC [13, 14] nomograms. It should be noted that these nomograms were developed and validated in populations differing in pre-biopsy mpMRI availability, biopsy technique (systematic vs. MRI-targeted), ethnic background, and prostate cancer risk group distribution, factors that may affect their predictive performance [15–17].

In prostate cancer, ML models contribute to early diagnosis and the development of personalized treatment plans by providing more accurate results based on patients’ individual characteristics [18].

In recent years, as in many areas of medicine, ML-based studies have gained momentum in uro-oncology. Numerous ML-based models have been developed in the literature to predict lymph node invasion (LNI). These models can offer higher accuracy rates compared to traditional nomograms and have the potential to reduce false-positive rates. For example, the PLNM-Risk calculator has demonstrated high accuracy with an AUC value of 93% and has been effective in reducing false-positive rates [19].

To the best of our knowledge, there is no ML-based study in the literature that simultaneously evaluates the Briganti Nomogram, Partin Nomogram, and MSKCC Nomogram. In this study, we aimed to compare the predictive values of existing nomograms with the ML-based models we developed for predicting the risk of lymph node invasion (LNI) in prostate cancer.

## Materials and methods

### Data analysis and machine learning

Patients recorded in the prospectively maintained “Çukurova University Faculty of Medicine Prostate Cancer Database” were retrospectively reviewed. The database covers prospectively collected data from January 2011 to June 2024. Patients who did not undergo lymph node dissection or had incomplete data were excluded from the analysis. The study included various demographic, clinical, and pathological variables. The database also contained scores calculated using the Partin, Briganti, and MSKCC (Memorial Sloan Kettering Cancer Center) nomograms. These scores were compared with prediction models developed using the dataset variables. For the Briganti nomogram, we applied the 2012 updated version [10]. Although mpMRI-derived variables (e.g., PIRADS score) were available in our database, the 2017 and 2019 mpMRI-integrated formulas were not used, as key parameters such as lesion size and MRI-specific risk weights required for those updates were not incorporated in our calculation. In prediction-based studies, the evaluation metrics used to assess model performance are of critical importance.

The dataset was divided into training (70%) and test (30%) subsets. Logistic Regression, SVMs, Random Forest, and Neural Networks were evaluated using 10-fold cross-validation, with AUC-ROC selected as the primary performance metric due to its threshold-independent nature. Hyperparameters were optimized for selected models (e.g., KNN with 5 neighbors, Random Forest via RandomizedSearchCV, Linear SVM with C = 0.025 and class_weight="balanced,” RBF SVM with gamma = 1, and MLP with alpha = 1 and max_iter = 1000), while the remaining algorithms were run with default parameters.

In this study, model performance was primarily assessed using the Area Under the Receiver Operating Characteristic Curve (AUC-ROC), which provides a comprehensive measure of discriminatory ability. To ensure statistical robustness, 95% confidence intervals (CIs) for the AUC values were calculated, allowing for an evaluation of the precision and reliability of the performance estimates. The AUC, a widely used metric in classification tasks, ranges from 0 to 1 and reflects the overall ability of a model to correctly distinguish between classes.

A total of 26 features were used in model development, including 4 demographic, 7 clinical, 6 laboratory, and 9 histopathological parameters. These features included age, BMI, smoking status, presence of diabetes mellitus, D’Amico risk group, PSA density, clinical stage, sPSA, total PSA, sPSA/total PSA ratio, ISUP grade, perineural invasion, lymphovascular invasion, positive core count, negative core count, percentage of positive cores, PIRADS score, and the MSKCC, Briganti, and Partin scores. The predictive value of these features in estimating lymph node invasion (LNI) was evaluated using both conventional nomograms and machine learning algorithms. Although body mass index (BMI) and diabetes mellitus are not direct predictors of lymph node invasion (LNI), they were included as surrogate markers of metabolic and hormonal status that may influence prostate cancer aggressiveness and potentially enhance the overall performance of the predictive model [20, 21]. Both systematic and MRI-targeted biopsies were performed in the same session when indicated, and all cores—including those obtained from the same MRI target—were pooled and recorded as a single dataset for analysis. Positive core count was defined as the total number of biopsy cores containing histologically confirmed prostate adenocarcinoma. Negative core count was defined as the total number of biopsy cores without evidence of malignancy. In patients undergoing combined systematic and MRI-targeted biopsies, both systematic and MRI-targeted cores were included in these counts.

**Fig. 1:**
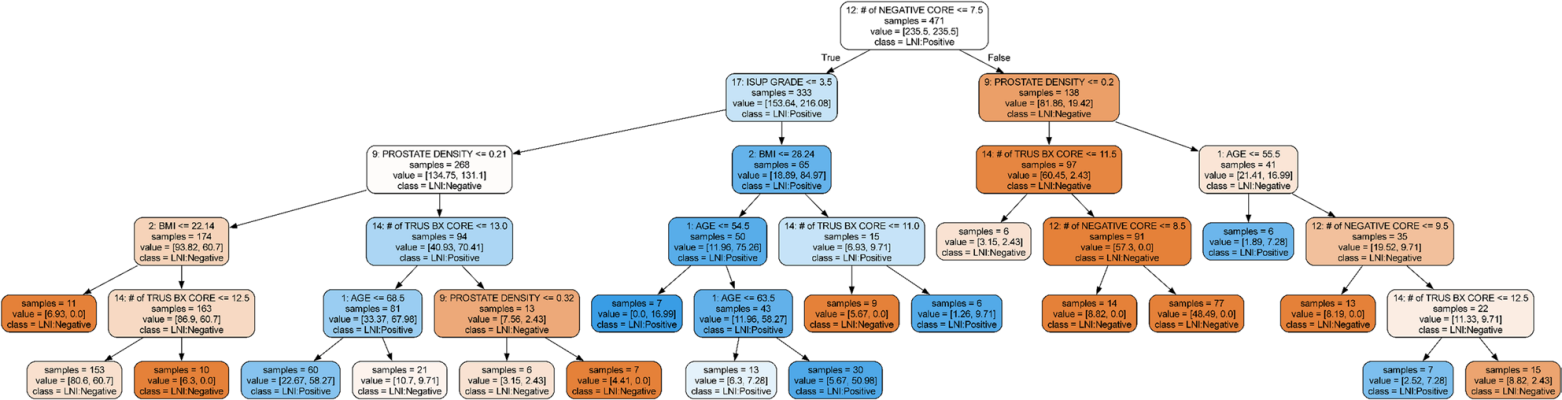
Decision Tree Constructed Using the Most Important Feature Subset (Our Model). (Abbreviations: ISUP grade: International Society of Urological Pathology grade / BMI: Body Mass Index / TRUS BX CORE: number of biopsy cores obtained via transrectal ultrasound-guided prostate biopsy / PROSTATE DENSITY: PSA density / AGE: patient age (years) / NEGATIVE CORE: number of biopsy cores without malignancy / LNI: Lymph Node Involvement.)

To comprehensively evaluate predictive performance, eleven ML algorithms were implemented, including Logistic Regression, K-Nearest Neighbors (KNN), Linear Support Vector Machine (SVM), Radial Basis Function (RBF) SVM, Decision Tree, Random Forest (RF), Multilayer Perceptron (MLP), Gaussian Process, AdaBoost, Gaussian Naïve Bayes, and Quadratic Discriminant Analysis. For the Decision Tree classifier, hyperparameter optimization was conducted using the GridSearchCV approach, and subsequently, a decision tree was constructed based on the most informative subset of features (Fig. 1).

Receiver Operating Characteristic (ROC) curves and the corresponding Area Under the Curve (AUC) values were utilized to assess the discriminatory ability of the models. Unlike single-threshold performance metrics such as accuracy or F1-score, the AUC-ROC metric evaluates model performance across the entire range of decision thresholds, thereby providing a more comprehensive and robust assessment. This characteristic is particularly advantageous in the context of imbalanced datasets, where reliance on single-threshold metrics may lead to misleading interpretations. By summarizing the trade-off between sensitivity and specificity over all possible thresholds, AUC-ROC offers a more reliable indication of the clinical utility and generalizability of the models.

There was a significant class imbalance in the original dataset. To address this imbalance, resampling techniques such as SMOTE (Synthetic Minority Over-sampling Technique), ADASYN (Adaptive Synthetic), Random Oversampling, and SMOTE-Tomek Link were applied exclusively during the training phase (Table 1).

**Table 1:** Best-Performing Models and Evaluation Metrics for Original and Resampled Datasets

Dataset	Model	ROC-AUC (95% CI)
Original (No resampling)	Neural Net	0.81 (0.75–0.87)
SMOTE	AdaBoost	0.73 (0.62–0.82)
SMOTE-Tomek Link	AdaBoost	0.79 (0.70–0.86)
ADASYN	Random Forest	0.75 (0.63–0.85)
Random Oversampling	Random Forest	0.73 (0.63–0.82)

Table 1 presents the best-performing model for each dataset configuration along with their evaluation metrics

ML models were developed using Python programming language version 3.11 (Python Software Foundation, 2023) [22] and PyCharm IDE version 2024.3(JetBrains, 2023) [23]. Implementation of ML algorithms was performed using the Scikit-learn library version 1.6 [24].

Within the scope of the study, the predictive performance of the Briganti, Partin, and MSKCC scores in estimating lymph node invasion was evaluated using the aforementioned ML models, both individually and in combination. Additionally, the performance of the custom-developed model was assessed independently and compared with the accuracy of these three classical scoring systems.

### Pathological evaluation

Histopathological examinations were performed by an experienced uropathologist in the Department of Pathology at our institution. Prostate volume was measured using transabdominal ultrasonography, and prostate-specific antigen (PSA) density was calculated by dividing the serum PSA value by the measured prostate volume. Clinical T staging was determined based on findings from digital rectal examination. All patients underwent laparoscopic radical prostatectomy. Tumor grading was carried out according to the 2014 ISUP/WHO criteria. For each biopsy, the total number of cores obtained, the positive core count (number of cores containing histologically confirmed prostate adenocarcinoma), and the negative core count (number of cores without evidence of malignancy) were recorded. Lymphovascular and perineural invasion were assessed on standard hematoxylin–eosin–stained formalin-fixed paraffin-embedded tissue sections. ISUP grade, PIRADS score, and lymphovascular invasion status were incorporated as independent variables in both the statistical analyses and the machine-learning models. These pathological data were used both to calculate nomogram scores (Briganti, Partin, and MSKCC) and as input variables for the machine-learning models developed to predict lymph-node invasion.

### Statistical analysis methods

Statistical analyses were conducted using IBM SPSS Statistics for Windows, Version 26.0 (Armonk, NY: IBM Corp.). The distribution of data was assessed using the Kolmogorov-Smirnov test. For continuous variables with normal distribution, Student’s t-test was used, while the Mann-Whitney U test was applied for non-normally distributed variables. Categorical variables were analyzed using Pearson’s chi-square test or Fisher’s exact test, as appropriate. A *p*-value < 0.05 was considered statistically significant. Additionally, ROC curves were generated, and AUC values were calculated for variables that reached statistical significance to evaluate their predictive performance. These statistical analyses were employed to support the results obtained from ML models.

### Ethical approval and study compliance

This study was conducted as a single-center investigation, with all data collected exclusively by physicians at the Department of Urology, Çukurova University Faculty of Medicine, Balcalı Hospital. Ethical approval was obtained from the Çukurova University Faculty of Medicine Institutional Review Board (Meeting No: 149, Date: 08.11.2024, Decision No: 69). The study was carried out in full compliance with all relevant ethical guidelines and regulations. The research procedures adhered to the ethical principles of the Declaration of Helsinki, ensuring the protection of participants’ health, privacy, and rights. Due to the retrospective nature of the study, the ethics committee determined that obtaining informed consent was not required. Consequently, no informed consent was obtained, and no direct or indirect contact with patients occurred, either in person or through communication tools (Appendix 1).

## Results

A total of 471 patients were included in the study; among them, 374 (79.4%) had no lymph node involvement, while 97 (20.6%) had lymph node involvement (Table 3).

**Table 2:** Demographic, Clinical, Laboratory, and Histopathological Data of Patient Groups

Variables	Lymph Node Involvement (-)	Lymph Node Involvement (+)	Total	*p*
Patient Count (*n*)	374(%79,4)	97(%20,6)	471(%100)	
Age(year) (*n*)	63,15 ± 7,07(374)	63,61 ± 7,63(97)	63,24 ± 7,18(471)	*0*,*676*
BMI* (*n*)	27,11 ± 3,08(374)	27,12 ± 3,29(97)	27,11 ± 3,12	*0*,*224*
Smoking Status(+) (%)				
Smoker	81(%21,7)	29(%29,9)	110(%23,4)	*0,166*
Non-Smoker	293(%78,3)	68(%70,1)	361(%76,6)	
DM** (+) (%)				
Yes	47(%12,6)	14(%14,4)	61(%13,0)	*0*,*200*
No	327(%87,4)	83(%85,6)	410(%87,0)	
*f*PSA *** (ng/mL) (*n*)	1,25 ± 0,62(73)	1,34 ± 0,71(12)	1,26 ± 0,63(85)	*0*,*322*
Total Psa (ng/mL) (*n*)	8,30 ± 3,40(374)	9,39 ± 3,53(97)	8,52 ± 3,45	*0*,*228*
sPSA/Total PSA (*n*)	0,13 ± 0,04 (72)	0,13 ± 0,04(12)	0,13 ± 0,04(84)	*0*,*547*
D’Amico Risk Group				
Low	121(%32,4)	11(%11,3)	132(%28,0)	*0*,*067*
Intermediate	108(%28,9)	20(%20,6)	128(%27,2)	
High	84(%22,5)	39(%40,2)	123(%26,1)	
Locally Advanced	61(%16,2)	27(%27,9)	88(%18,7)	
PSA density (ng/mL/cc)	0,19 ± 0,16(374)	0,26 ± 0,19(97)	0,20 ± 0,17(471)	*0*,*237*
Clinical Stage				
T1a	1(%0,3)	2(%2,1)	3(%0,6)	*0*,*583*
T1b	12(%3,2)	1(%1)	13(%2,6)	
T1c	190(%50,8)	29(%29,9)	219(%44,4)	
T2a	57(%15,2)	17(%17,5)	74(%15,0)	
T2b	11(%2,9)	9(%9,3)	20(%4,1)	
T2c	55(%14,7)	17(%17,5)	72(%14,6)	
T3a	31(%8,4)	8(%8,2)	39(%7,9)	
T3b	17(%4,5)	12(%12,4)	29(%5,9)	
T4	0(%0,0)	2(%2,1)	2(%0,4)	
Positive Core Count (*n*)****	4,74 ± 3,25 (374)	4,31 ± 3,20 (97)	4,64 ± 3,22(471)	*0*,*302*
Negative Core Count (*n*)****	5,77 ± 2,77 (374)	4,46 ± 2,51 (97)	5,58 ± 2,73(471)	*0*,*942*
Positive Core Percentage (*n*)	41,61 ± 24,16(374)	47,64 ± 27,59(97)	42,91 ± 24,98(471)	*0*,*454*
Core Count (*n*)	12,56 ± 7,19(374)	9,58 ± 4,96 (97)	11,94 ± 6,85(471)	*0*,*772*
Perineural Invasion (+) (%)				
Yes	35(%9,4)	8(%8,2)	43(%9,1)	*0*,*150*
No	339(%90,6)	89(%91,8)	428(%90,9)	
Lymphovascular Invasion (+) (%)				
Yes	6(%1,6)	1(%1)	7(%1,5)	*0,027*
No	368(%98,4)	96(%99)	464(%98,5)	
ISUP Grade*****				
1	231(%61,8)	40(%41,2)	271(%57,5)	*0,007*
2	60(%16)	12(%12,4)	72(%15,3)	
3	40(%10,7)	9(%9,3)	49(%10,4)	
4	28(%7,5)	21(%21,6)	49(%10,4)	
5	15(%4,0)	15(%15,5)	30(%6,4)	
PIRADS Score				
1	1(%3,6)	0(%0,0)	1(%2,4)	*0,014*
2	2(%7,1)	2(%15,4)	4(%9,8)	
3	4(%14,3)	0(%0)	4(%9,8)	
4	8(%28,6)	5(%38,5)	13(%31,7)	
5	13(%46,4)	6(%46,2)	19(%46,3)	
PSMA-PET Lymph Node Involvement (+) (%)				
Yes	16(%50)	10(%52,6)	26(%51,0)	*0,048*
Mo	16(%50)	9(%47,4)	25(%49,0)	
MSKCC skore (*n*)	4,02 ± 2,03 (374)	4,50 ± 1,61 (97)	4,12 ± 1,96(471)	*0,027*
Briganti skore (*n*)	6,40 ± 5,08 (374)	9,52 ± 5,06 (97)	7,04 ± 5,23(471)	*0*,*051*
Partin skore (*n*)	2,22 ± 1,88 (374)	2,67 ± 1,39 (97)	2,31 ± 1,80(471)	*0*,*020*

*BMI Body Mass Index /

**DM Diabetes Mellitus /

***fPSA Free Prostat Spesifik Antijen

**** Positive core count: total number of biopsy cores positive for adenocarcinoma. Negative core count: total number of biopsy cores without malignancy. Both systematic and MRI-targeted cores were included

*****All variables listed include combined data from systematic and MRI-targeted biopsies

There were no statistically significant differences between the two groups with respect to age (*p* = 0.676), body mass index (*p* = 0.224), smoking status (*p* = 0.166), presence of diabetes mellitus (*p* = 0.200), fPSA level (*p* = 0.322), total PSA level (*p* = 0.228), sPSA/total PSA ratio (*p* = 0.547), D’Amico risk group (*p* = 0.067), PSA density (*p* = 0.237), clinical stage (*p* = 0.583), positive and negative biopsy core counts (*p* = 0.302 and *p* = 0.942, respectively), percentage of positive cores (*p* = 0.454), total core count (*p* = 0.772), and Briganti score (*p* = 0.051) (all *p* > 0.05) (Table 3).

In contrast, lymphovascular invasion (*p* = 0.027), ISUP grade (*p* = 0.007), PIRADS score (*p* = 0.014), PSMA-PET–detected lymph node involvement (*p* = 0.048), MSKCC score (*p* = 0.027), and Partin score (*p* = 0.020) demonstrated statistically significant differences between patients with and without lymph node involvement (all *p* < 0.05) (Table 3).

The predictive performance of the ML algorithms and the three nomograms (MSKCC, Briganti, and Partin) was evaluated using AUC values. Overall, the nomograms demonstrated moderate discrimination, with AUCs in a similar range. Across individual and combined analyses, several ML models achieved AUC values that were numerically comparable to those of the nomograms, with the highest observed AUCs seen in Neural Network, Gaussian Naive Bayes, and AdaBoost models, depending on the feature set used.

**Fig. 2:**
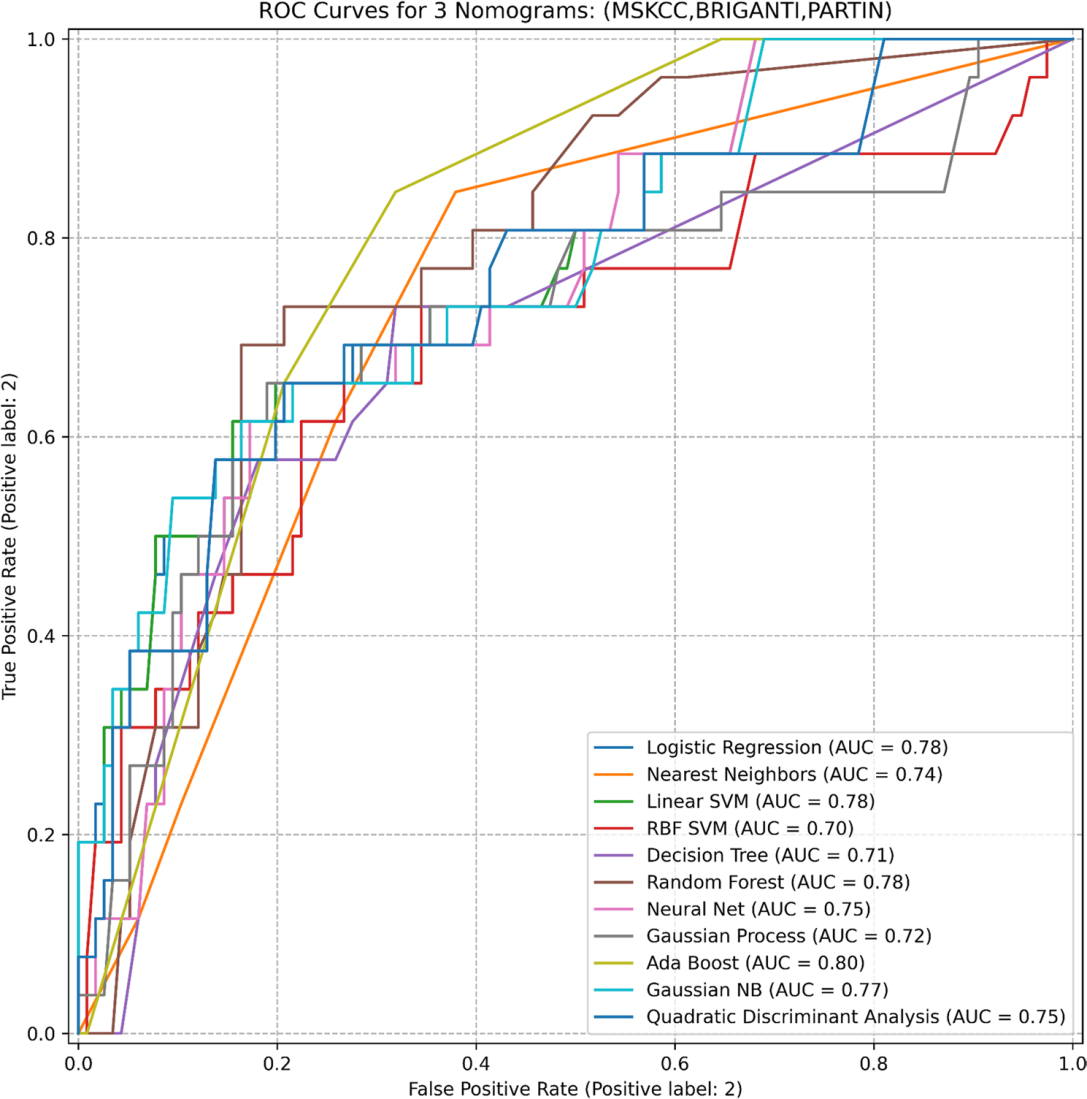
Results of the Three Nomograms. This figure illustrates the ROC curves for eleven different machine learning classifiers used to predict LNI. The area under the curve (AUC) values indicate the predictive performance of each model, with higher AUC values representing better discrimination ability. (Abbreviations: ROC, receiver operating characteristic; AUC, area under the curve; MSKCC, Memorial Sloan Kettering Cancer Center; SVM, support vector machine; RBF, radial basis function; NB, Naive Bayes)

**Fig. 3:**
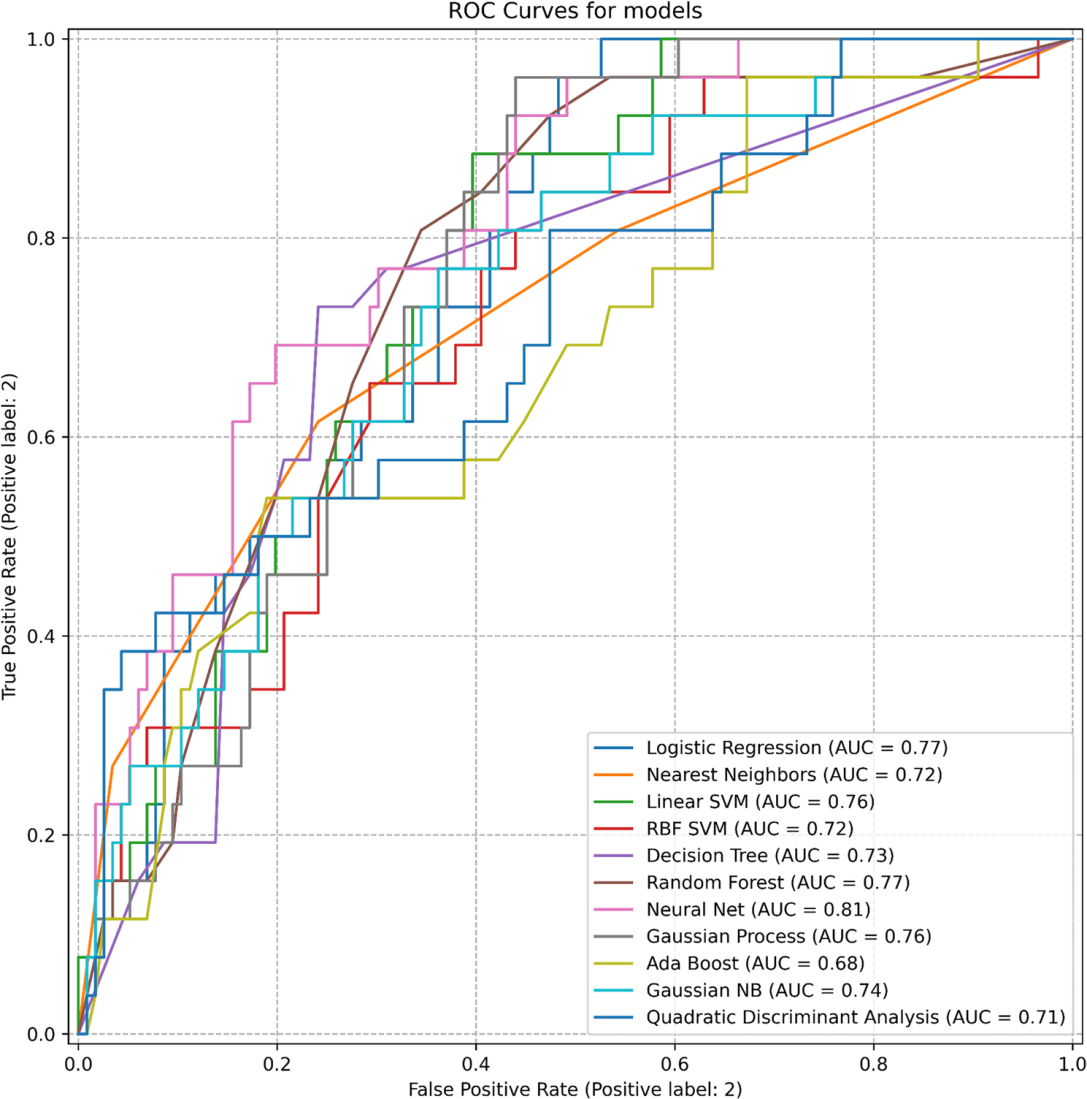
Results of Our Model. Receiver operating characteristic (ROC) curves illustrating the performance of multiple machine-learning models for predicting lymph node involvement. The area under the curve (AUC) is shown for each classifier: Logistic Regression (AUC = 0.77), Nearest Neighbors (AUC = 0.72), Linear SVM (AUC = 0.76), RBF SVM (AUC = 0.72), Decision Tree (AUC = 0.73), Random Forest (AUC = 0.77), Neural Net (AUC = 0.81), Gaussian Process (AUC = 0.76), Ada Boost (AUC = 0.68), Gaussian NB (AUC = 0.74), and Quadratic Discriminant Analysis (AUC = 0.71). (Abbreviations: ROC, receiver operating characteristic; AUC, area under the curve; SVM, support vector machine; RBF, radial basis function; NB, Naive Bayes.)

**Fig. 4:**
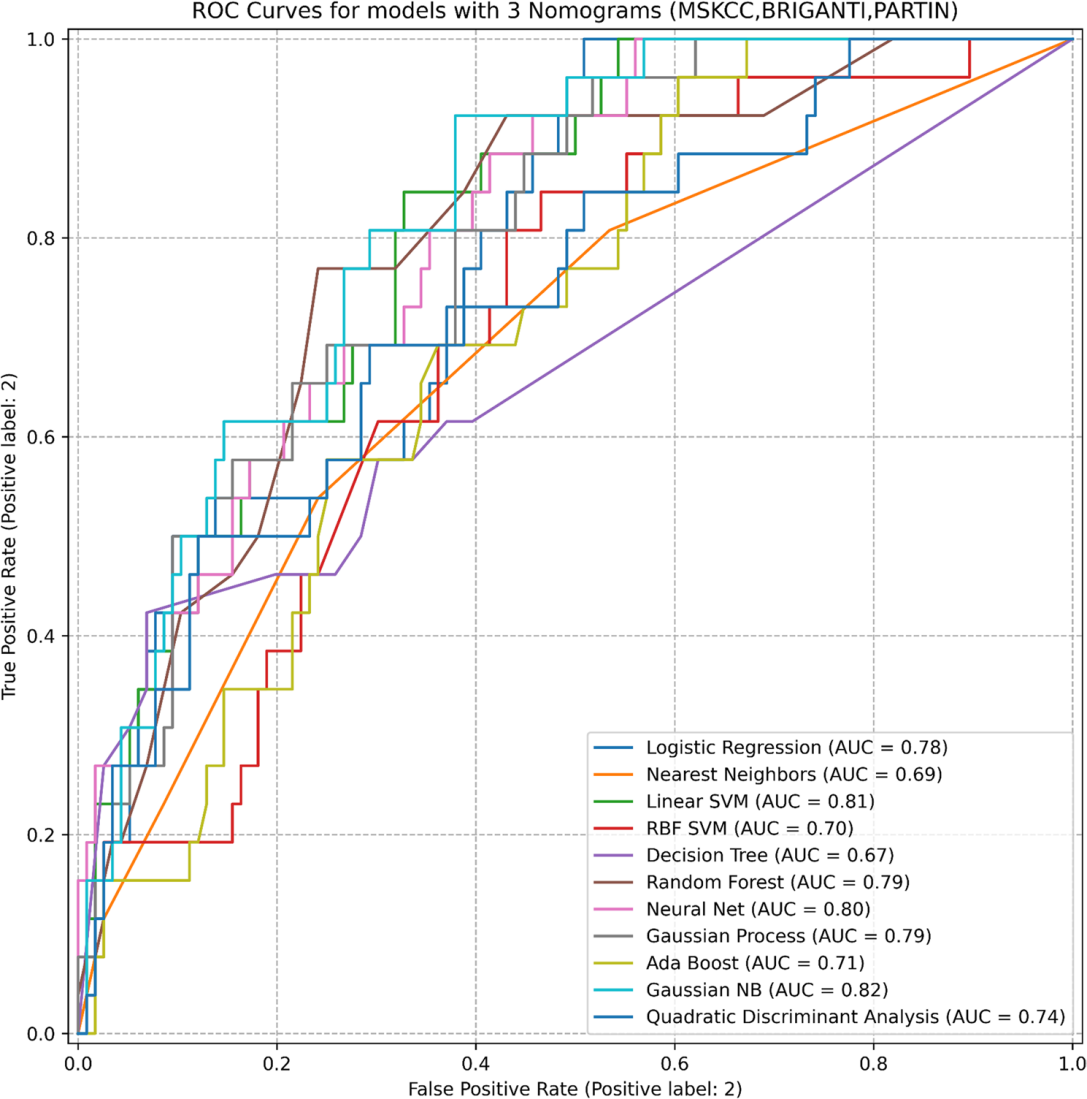
Results of Our Model with Three Nomograms. ROC curves of machine-learning models combined with three nomograms (MSKCC, Briganti, Partin) for predicting lymph node involvement. AUC values: Logistic Regression 0.78, Nearest Neighbors 0.69, Linear SVM 0.81, RBF SVM 0.70, Decision Tree 0.67, Random Forest 0.79, Neural Net 0.80, Gaussian Process 0.79, Ada Boost 0.71, Gaussian NB 0.82, Quadratic Discriminant Analysis 0.74. (Abbreviations: ROC, receiver operating characteristic; AUC, area under the curve; SVM, support vector machine; NB, Naive Bayes.)

In models incorporating combined nomogram scores or all available variables, modest numerical increases in AUC were observed; however, performance estimates largely overlapped across models. Detailed AUC values and confidence intervals for individual models and combinations are presented in Tables 1 and 3; Figs. 2, 3 and 4.

**Table 3:** Models and Evaluation Metrics for various scenarios

Dataset	Model	ROC-AUC (95% CI)
3 Nomograms	Logistic Regression	0.78 (0.6272–0.9326)
	Nearest Neighbors	0.74 (0.6321–0.8515)
	Linear SVM	0.78 (0.6334–0.9366)
	RBF SVM	0.70 (0.5677–0.8418)
	Decision Tree	0.71 (0.5761–0.8437)
	Random Forest	0.78 (0.6450–0.9222)
	Neural Net	0.75 (0.5944–0.9014)
	Gaussian Process	0.72 (0.5685–0.8798)
	Ada Boost	0.80 (0.6529–0.9404)
	Gaussian NB	0.77 (0.6064–0.9359)
	Quadratic Discriminant Analysis	0.75 (0.5791–0.9158)
Our Model	Logistic Regression	0.77 (0.7062–0.8337)
	Nearest Neighbors	0.72 (0.6216–0.8189)
	Linear SVM	0.76 (0.7125–0.8114)
	RBF SVM	0.72 (0.6991–0.7507)
	Decision Tree	0.73 (0.6249–0.8281)
	Random Forest	0.77 (0.6657–0.8687)
	Neural Net	0.81 (0.7502–0.8706)
	Gaussian Process	0.76 (0.6508–0.8646)
	Ada Boost	0.68 (0.5927–0.7624)
	Gaussian NB	0.74 (0.6638–0.8155)
	Quadratic Discriminant Analysis	0.71 (0.6056–0.8063)
Our Model with 3 Nomograms	Logistic Regression	0.78 (0.7061–0.8473)
	Nearest Neighbors	0.69 (0.6139–0.7640)
	Linear SVM	0.81 (0.7453–0.8706)
	RBF SVM	0.70 (0.6736–0.7257)
	Decision Tree	0.67 (0.5862–0.7447)
	Random Forest	0.79 (0.6752–0.9059)
	Neural Net	0.80 (0.6868–0.9142)
	Gaussian Process	0.79 (0.6887–0.8955)
	Ada Boost	0.71 (0.5938–0.8280)
	Gaussian NB	0.82 (0.7208–0.9228)
	Quadratic Discriminant Analysis	0.74 (0.6591–0.8227)

**Fig. 5:**
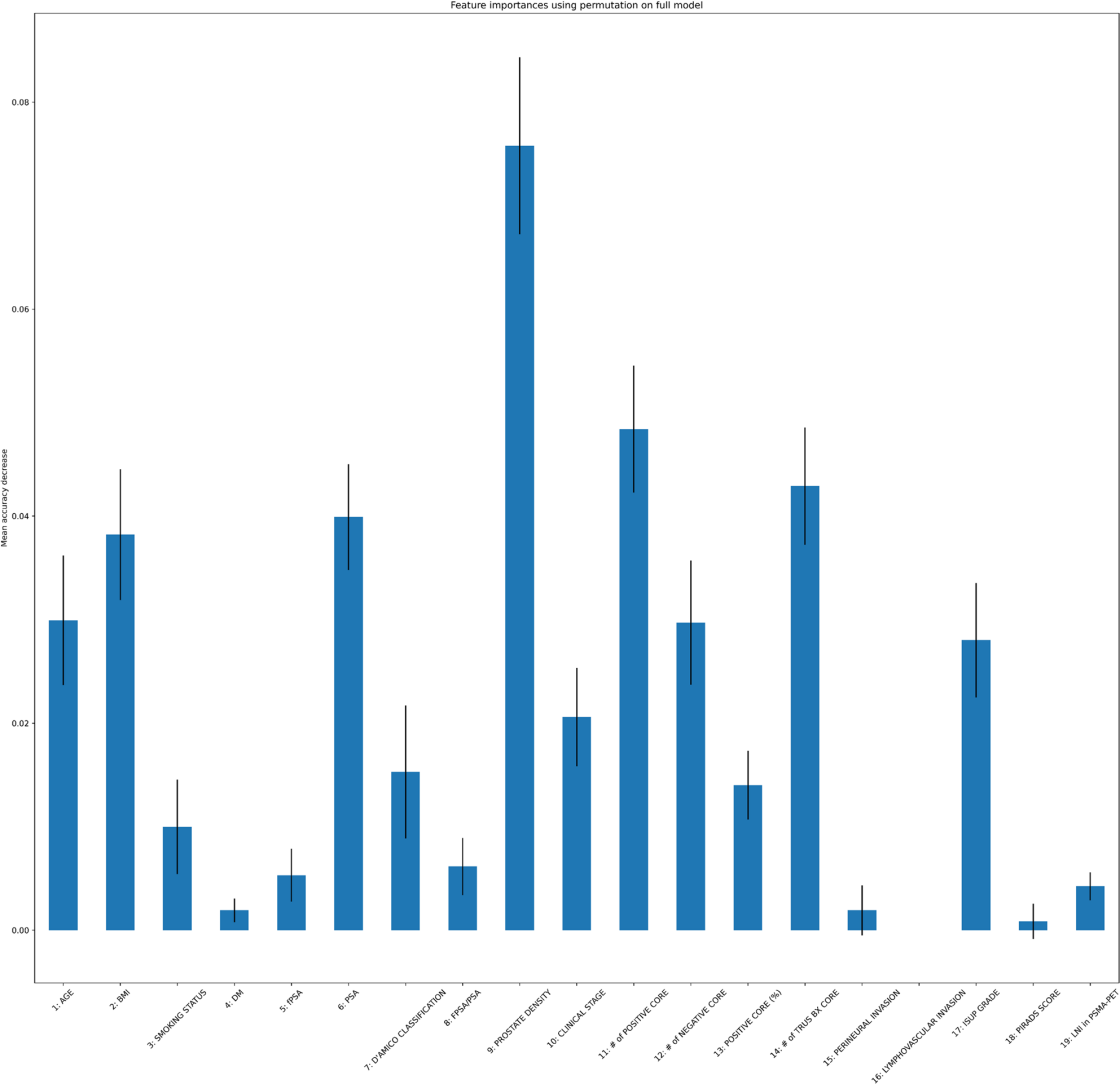
Evaluation of Feature Importance with the Random Forest Model. Feature importance of clinical and pathological variables estimated by permutation on the full prediction model. Bars represent the mean decrease in model accuracy when the feature is permuted, with error bars indicating standard deviation. (Abbreviations: PSA, prostate-specific antigen; ISUP grade, International Society of Urological Pathology grade; TRUS BX CORE, transrectal ultrasound-guided biopsy core count; LNI, lymph node involvement.)

Data-balancing strategies did not lead to consistent performance improvements. Among resampling approaches, SMOTE-Tomek yielded the highest AUC values, although these remained within a similar range to those obtained from the original dataset (Table 1). Feature importance analyses identified prostate density, biopsy core counts, PSA, ISUP grade, BMI, and age as influential predictors (Fig. 5). Decision-tree analysis further demonstrated clinically interpretable thresholds—particularly negative biopsy core count, ISUP grade, and prostate density—that stratified LNI risk into distinct groups (Fig. 1).

Classical statistical analyses supported the model-based findings, showing significant associations between LNI and ISUP grade, PIRADS score, lymphovascular invasion, PSMA PET–detected nodal involvement, and MSKCC and Partin scores, while no significant differences were observed for other evaluated variables (Table 3).

## Discussion

In this study, the performance of the machine learning-based model developed to predict lymph node invasion (LNI) prior to radical prostatectomy was compared with the Partin, Briganti, and MSKCC nomograms.

Our findings suggest that the predictive accuracy of the machine learning model was comparable to that of traditional nomograms, although the current study design may limit the generalizability of these results. According to the literature, the Briganti nomogram has been reported to provide high accuracy with an AUC of 88% [25], while the MSKCC nomogram reached an accuracy rate of 79% in patients with Gleason scores ≥ 8 [26]. Additionally, the Partin nomogram has been shown to be effective in predicting lymph node involvement and seminal vesicle invasion [27]. Furthermore, these three nomograms have been demonstrated to provide similar accuracy in predicting LNI [28].

ML methods have increasingly become a valuable tool in clinical decision-making within the field of urology, as in other areas of healthcare. In addition to oncological applications, ML is also effectively used in other subfields of urology, including stone disease [29], infertility [30], urinary tract infections [31], and incontinence [32].

ML is being effectively applied across various disciplines due to its superior ability to analyze complex data structures and make accurate predictions compared to traditional statistical methods. In the healthcare sector, ML significantly contributes to early diagnosis and treatment by providing higher accuracy and reliability, particularly in medical diagnostic processes [33].

In this study, the MSKCC, Briganti, and Partin nomograms showed moderate performance in predicting lymph node invasion (AUCs: 0.79, 0.77, and 0.78, respectively). Overall, ML algorithms showed comparable performance to the nomograms; when used alone, the Neural Net achieved the highest observed AUC (0.81). However, the statistical significance of between-model AUC differences was not formally compared in this study. In the analysis of datasets balanced using SMOTE techniques, SMOTE-Tomek emerged as the most effective method; however, its performance did not surpass that achieved on the imbalanced dataset. Combining nomogram scores improved performance, with AdaBoost reaching an AUC of 0.80. The most comprehensive model incorporating all variables yielded numerically higher AUC values; however, overall performance largely overlapped with that of established nomograms, suggesting comparable rather than superior discrimination. These findings indicate performance similarity between ML-based models and traditional nomograms rather than a clear incremental improvement.

In a study by Hou et al. (2019), a machine learning–assisted model was developed to predict the necessity of ePLND in patients with prostate cancer. When combined with MRI data, the model achieved an AUC of 0.906, surpassing the performance of the MSKCC nomogram (AUC 0.816). Decision tree analysis revealed that the model primarily relied on MRI-based predictions of lymph node invasion (LNI) and seminal vesicle invasion (SVI), followed by PSA density and the number of positive biopsy cores to stratify risk groups [34].

Wang et al. (2023) demonstrated that ML models possess higher predictive accuracy than conventional clinical nomograms in estimating lymph node metastasis in prostate cancer (ML: C-index 0.862; Briganti: 0.745; MSKCC: 0.714). The most important predictive features were identified as PSA level, Gleason score, and clinical T stage. The study emphasized that integrating ML models with nomograms could lead to more effective and accurate predictive outcomes [35].

In a study conducted by Semwal et al. (2024), ML–based models, including Extreme Gradient Boosting (XGBoost), Logistic Regression, Random Forest, and Support Vector Machine (SVM), were evaluated for predicting pathological stage in patients with localized prostate cancer. XGBoost outperformed traditional nomograms (MSKCC, Briganti, and Partin) in predicting organ-confined disease (AUC: 0.744), extracapsular extension (AUC: 0.749), seminal vesicle invasion (AUC: 0.816), and lymph node involvement (AUC: 0.811). The most important predictive features identified were the percentage of positive biopsy cores, PSA level, Gleason score, and clinical T stage [36].

Feature importance analysis for the Random Forest model revealed several variables with substantial contributions to predicting lymph node invasion (LNI). Among these, prostate-specific antigen (PSA) levels, prostate density, clinical stage, percentage of positive cores, and age emerged as the most influential features. This highlights the critical role of both clinical and pathological parameters in LNI prediction. The Random Forest model, optimized with parameters of 30 estimators, a maximum depth of 50, a minimum sample split of 2, and the square root criterion for maximum features, demonstrated robust performance while maintaining interpretability through these feature importance insights. These findings emphasize that incorporating key clinical variables significantly enhances model accuracy and may guide more informed preoperative decision-making in clinical practice. These findings are consistent with the key predictors emphasized in other studies in the literature [35, 37]. Our Neural Net–based ML model, through multidimensional analysis of clinical and pathological parameters, demonstrated at least comparable discrimination than existing nomograms; external validation and additional comparative analyses are needed to establish the statistical and clinical significance of these differences.Moreover, it holds significant potential for guiding adjuvant treatment decisions following radical prostatectomy by supporting more accurate staging.

The decision tree analysis constructed using the most significant variables in our model (Fig. 1) revealed clinically relevant threshold values that effectively stratify the risk of lymph node invasion (LNI). In the constructed decision tree model, “number of negative core” emerged as the root node, indicating it provides the highest information gain and serves as the primary criterion for data partitioning. Immediately following the root, “isup grade” and “prostate density” were identified as the most influential features, with “isup grade” representing the second most decisive factor in the hierarchy. At subsequent levels, “prostate density,” “body mass index,” “number of trus bx core,” and “age” collectively contributed to further splits, thereby improving the model’s discriminative performance. In alternative branches, “body mass index,” “number of trus bx core,” and “age” consistently appeared as relevant predictors, emphasizing their sustained influence on decision-making. Based on our decision tree model, ISUP Grade > 3, Prostate Density > 0.2, BMI > 22.14, Age > 54.5, a number of Negative Cores < 7.5–9.5, and a number of TRUS-Bx Cores > 11.0–13.0 emerged as the most influential parameters and were identified as high-risk factors for lymph node invasion. Overall, this hierarchical structure underscores the robust predictive importance of “prostate density” and “body mass index” within the model.

One of the key advantages of the developed decision tree model is its ability to provide a rapid and interpretable classification of LNI risk. By integrating clinical, biochemical, and pathological parameters, the model supports personalized patient management. The root node—positive biopsy core count—was identified as the most influential variable, with increasing values strongly associated with elevated LNI risk. Other contributing features such as total biopsy count, PSA level, MSKCC score, age, Partin score, and PIRADS score allowed for layered stratification across clinically meaningful cut-off values. Particularly, patients with PSA > 12 ng/mL and MSKCC score > 6 were predicted to have high LNI risk. Moreover, younger patients (≤ 55.5 years) with high-risk biopsy characteristics—such as low negative core count and high PSA density—also demonstrated increased LNI probability.

In our study, PSA density, negative core count, PSA level, and ISUP grade were significantly associated with LNI. Furthermore, parameters such as PIRADS score, lymphovascular invasion, MSKCC score, and Partin score—identified as significant in both traditional statistical analysis and the ML model—support the clinical validity of our findings.

Although PIRADS scores in our cohort were relatively low, this likely reflects the retrospective design and heterogeneous imaging practice, where mpMRI was not routinely performed with a standardized protocol and was available mainly in lower-risk cases [38]. Importantly, PIRADS was integrated with other clinical and pathological variables in the ML models, thereby minimizing the impact of its lower distribution on overall predictive performance. Similarly, although PSMA PET/CT provides high sensitivity for primary staging, contemporary evidence and EAU 2025 guidelines emphasize that it should not be used as a sole determinant of lymph-node invasion (LNI). In our study, PSMA PET findings were integrated with clinical and pathological variables within the machine-learning models rather than being used as an isolated predictor, in line with current recommendations and recent evidence [4, 11]. In addition, PSMA PET/CT offers important advantages, such as its ability to detect small lymph node metastases with high sensitivity and specificity, to provide complementary information in the staging process, and to contribute to more accurate risk stratification in treatment planning. On the other hand, heterogeneity in imaging protocols, inter-observer variability in interpretation, issues of cost and accessibility, limitations in detecting very small lesions, and physiological PSMA uptake leading to false-positive findings have been emphasized in the literature as major limitations of this modality.

In our study, absolute positive and negative core counts were stronger predictors of lymph-node invasion (LNI) than the percentage of positive cores. Unlike percentages, absolute counts are less affected by variation in total biopsy cores and provide a more direct measure of tumor burden and tumor-free tissue, yielding a clearer biological signal. Consistently, Random Forest and decision-tree analyses ranked these counts higher than the positive-core percentage (Figs. 1 and 3). These findings are in line with the updated Briganti nomogram (2012 and 2019), which likewise highlights the superior prognostic value of absolute positive core count [10, 11].

This study introduces an interpretable ML approach for preoperative pLNI prediction that demonstrates performance comparable to existing nomograms.Furthermore, a notable proportion of our cohort consisted of ISUP Grade Group 1 and D’Amico low-risk patients—who are not routinely recommended for pelvic lymph-node dissection (PLND) according to EAU 2025 guidelines—yet their inclusion was intentional to evaluate model robustness across the entire clinical spectrum and to demonstrate the model’s ability to safely identify candidates in whom PLND can be avoided.

### Limitations

This study has several limitations. First, it is a single-center, retrospective analysis based on the Çukurova University Prostate Cancer Database (January 2011–June 2024), which may restrict external generalizability and does not capture inter-institutional differences in patient selection, imaging workflows, or surgical technique. Second, imaging and biopsy parameters were not fully standardized across all cases: pre-biopsy mpMRI availability was heterogeneous, PIRADS scores were less frequently available and skewed toward lower values, PSMA PET/CT was acquired and interpreted under non-uniform protocols, and systematic and MRI-targeted biopsy cores were pooled—factors that could influence the performance of imaging-based predictors and core-derived features. Third, although we compared three widely used tools (Partin, Briganti [2012 version], and MSKCC), other contemporary models (e.g., Amsterdam–Brisbane–Sydney) were not evaluated because required inputs were not consistently available and, at the time of study design, these models had limited multi-institutional external validation and were not embedded in major guideline algorithms. Fourth, model development relied on internal validation (stratified train/test split with cross-validation for tuning); lack of prospective, multi-center external validation raises the possibility of optimism and limits transportability to different practice settings. In line with similar decision-tree or nomogram studies, external validation was not feasible in the present analysis, largely due to ethical constraints and the practical difficulty of accessing external institutional datasets. This limitation is common in the field and explains why only a minority of comparable studies are able to incorporate true external validation. Fifth, the class distribution (LNI prevalence ≈ 20%) and retrospective data capture may introduce residual confounding and missingness that we could not fully eliminate despite predefined inclusion criteria. Future work should pursue prospective, multi-center external validation with standardized mpMRI and PSMA PET protocols, assess additional nomograms using harmonized variable sets, and quantify clinical utility with decision-curve and net-benefit analyses.

## Conclusion

In conclusion, in this cohort, the AUC values of the evaluated ML models were numerically similar to those of the established nomograms (MSKCC, Briganti, and Partin), and in some comparisons only slightly higher. However, given the study’s inherent limitations—most notably its single-center retrospective design and the lack of external validation—these findings do not demonstrate a clinically reliable or generalizable advantage over existing nomograms. Therefore, traditional nomograms remain appropriate and clinically reliable tools for preoperative LNI risk estimation in current practice.

Decision-tree analysis identified clinically interpretable predictors (negative biopsy core count, ISUP grade, prostate density, PSA, BMI, and age), which may help describe risk patterns within this dataset. Future prospective multicenter studies with external validation are needed to confirm generalizability and to quantify clinical utility.

## Data Availability

The datasets generated and/or analyzed during the current study are available from the corresponding author upon reasonable request.

## Abbreviations

BMI: Body Mass Index
CT: Computed Tomography
EAU: European Association of Urology
ePLND: Extended Pelvic Lymph Node Dissection
fPSA: Free Prostate-Specific Antigen
ISUP: International Society of Urological Pathology
LND: Lymph Node Dissection
LNI: Lymph Node Invasion
mpMRI: Multiparametric Magnetic Resonance Imaging
MRI: Magnetic Resonance Imaging
PCa: Prostate Cancer
PET: Positron Emission Tomography
PIRADS: Prostate Imaging Reporting and Data System
pLNI: Pathological Lymph Node Invasion
PLND: Pelvic Lymph-Node Dissection
PSA: PSA
PSMA: Prostate-Specific Membrane Antigen
sPSA: Serum Prostate-Specific Antigen
SVI: Seminal Vesicle Invasion
TRUS-Bx: Transrectal Ultrasound Biopsy
WHO: World Health Organization
ABS: Amsterdam–Brisbane–Sydney
MSKCC: Memorial Sloan Kettering Cancer Center
ADASYN: Adaptive Synthetic
AUC: Area Under the Curve
AUC-ROC: Area Under the Receiver Operating Characteristic Curve
CI: Confidence Interval
IBM: International Business Machines
IDE: Integrated Development Environment
KNN: K-Nearest Neighbors
ML: Machine Learning
MLP: Multilayer Perceptron
RBF: Radial Basis Function
RF: Random Forest
ROC: Receiver Operating Characteristic
SMOTE: Synthetic Minority Over-sampling Technique
SPSS: Statistical Package for the Social Sciences
SVM: Support Vector Machine
XGBoost: Extreme Gradient Boosting
